# Indigenous practitioners’ views on causes of female infertility

**DOI:** 10.4102/hsag.v28i0.2152

**Published:** 2023-02-08

**Authors:** Banabotlhe G. Baakeleng, Abel J. Pienaar, Puledi M. Sithole, Simangaliso L. Mashego

**Affiliations:** 1 School of Nursing, Faculty of Health Sciences, North-West University, Mafikeng, South Africa; 2 Department of Nursing, Faculty of Nursing and Midwifery, Shifa Tameer-Millat University, Islamabad, Pakistan; 3 Department of Nursing, Faculty of Nursing and Midwifery, University of Venda, Thohoyandou, South Africa; 4 Indigenous Knowledge System Centre, Faculty of Agriculture and Natural Science, North-West University, Mafikeng, South Africa

**Keywords:** female, infertility, indigenous medicine, indigenous practitioner, African traditional medicine

## Abstract

**Background:**

The use of indigenous practices has increased remarkably throughout the world. Subsequently, society uses this practice for the treatment of various health problems, including infertility. This research focussed on the role of indigenous practitioners (IPs) using a holistic approach to explore the causes of infertility in women.

**Aim:**

This study aimed to explore and describe the views of IPs on the causes of female infertility in Ngaka Modiri Molema health district.

**Setting:**

The study was conducted in Ngaka Modiri Molema, North West Province, one of the most rural provinces in South Africa.

**Methods:**

The study followed a qualitative explorative design. A purposive sampling technique identified five IPs who were experts in managing infertility. Individual semi-structured interviews were conducted, and data analysis used Creswell’s method of qualitative data analysis.

**Results:**

Findings revealed that IPs offered a wide range of services in the treatment and management of infertility among rural women. Hence, the following themes emerged, namely, history taking regarding infertility, treatment of infertility and holistic care on infertility.

**Conclusion:**

The IPs are important providers of healthcare in the management of infertility in indigenous communities. The findings revealed that there are various causes of female infertility according to the indigenous healthcare system.

**Contribution:**

In contribution, the study described the unique practices found in the community as executed by the IPs. This care focusses on holistic care, including treatment and continuous care for the healthcare user and the family. Noteworthy to mention, this holistic care extends to subsequent pregnancies. However, there is a need for further research to valorise the indigenous knowledge unearthed in this study.

## Introduction and background

Globally, about 80% of the population in rural areas of developing countries seek help from indigenous practitioners (IPs) for all health challenges, including infertility, because it is accessible and inexpensive and has a variety of health benefits, such as few side effects (Jaradat & Zaid 2019:2). Infertility contributes to social and psychological problems of women because they face discrimination in some cultural groups and communities, which leads to stress. For example, according to Chinese culture, family is not just a social component, but rather an extension of the family line, and it is a violation of family trust if a woman is unable to procreate (Tiu et al. 2018:3). Some women with infertility choose to consult with IPs because they maintain privacy and provide holistic care (Kochhar et al. 2017:71). A quantitative study conducted by James et al. (2018:5) asserts that when infertility is associated with supernatural and spiritual causes, faith healing or visits to IPs are seen as a first-choice health-seeking behaviour. For example, consultation with IPs in Jordan is common and has increased remarkably to prevent causes of infertility and cure other reproductive problems (El-Dahiyat et al. 2020:1). Indigenous practitioners are widely trusted healthcare providers in the management of female infertility and related reproductive problems, such as uterine fibroids and hormonal imbalances (Shewamene, Dune & Smith 2017:2).

In Ethiopia, Kahissay, Fenta and Boon (2017:2) posited that IPs often believe that Western practitioners are not equipped to address women’s concerns that include causes of infertility and its management. A qualitative study conducted by Srishan et al. (2020:37) asserted that in Sri Lanka, IPs used modalities such as herbs, food and rituals since ancient times to manage and curb causes of infertility in women. Culture is a considerable factor that plays a significant role in people’s beliefs concerning diagnosis and causes of infertility, which results in accepting ritual activities prescribed by IPs (Ahmed, Yousaf & Khan 2017:3). It is crucial that healthcare practitioners, including the IPs, provide counselling and awareness campaigns on causes of infertility to promote uneventful conception and maintenance of pregnancy until birth. Some studies affirm that infertility is a reproductive issue that affects both sexes, but women are the ones blamed for childlessness (Chimbatata & Malimba 2016:97). Despite the availability of Western practice, women in Karnataka (India) still believe that infertility management encompasses cultural methods, and consequently, most women turn to various indigenous practices such as herbal concoctions and faith healing to curb infertility (Patil & Udgiri 2019:227).

Ofosu-Budu and Hänninen (2021:114) conducted a study in Ghana on the views of infertile women and their herbalists. Their findings revealed that sex education in schools is essential as it would prevent unhealthy sexual activities and unwanted pregnancies, which lead to illegal abortions that predispose women to infertility. The researcher has worked in a primary health setting where women with infertility reported back from the hospital that the reason for them not conceiving was unexplained, thus choosing to consult with an IP. Odmell, Mamimine and Chitindingu (2018:7) affirmed that in Zimbabwe, IPs remain in the forefront of illness control. In low-and-middle-income countries, inflated Western healthcare practices costs are beyond the reach of poor rural communities; however, the indigenous practice has continued to be the most affordable and easily accessible practice. According to Mdhluli (2019:64), IPs attest that infertility can be because of evil forces out of jealousy from ex-spouse, in-laws and other relatives. The same study affirmed that infertility is a punishment from God because of sinful deeds. Therefore, women consult with spiritual healers for prayers and preparation of holy water for cleansing curses that cause infertility; these women default treatment schedules with Western practices where the explanation regarding the causes of infertility do not align with their cultural beliefs (Mdhluli 2019:64; Ola, Aladekomo & Oludare 2008:193). The Western practice remains a challenge to many women suffering from infertility in Africa in terms of affordability, access and treatment failures (Tanywe et al. 2018:1773). Hence, the purpose of this article is to explore and describe IPs’ views on the causes of infertility in women.

Naab, Lawali and Donkor (2021:4) asserted that African women consult with Western practitioners for treatment of infertility, but may become dissatisfied and default on follow-up visits. Infertility is believed to be a public health problem that affects the well-being of women worldwide. The World Health Organization (WHO 2020) defined infertility as the ‘failure to conceive after 12 months of regular unprotected sexual intercourse in the absence of known reproductive pathology’. Infertility is the inability to achieve conception after at least 12 months of unprotected intercourse (Tanywe et al. 2018:1772). Causes of infertility require intense intervention to improve the fertility rate in North West Province, which is fluctuating as per records from Statistics South Africa (StatsSA). From 2011 to 2016, the fertility rate was 2.84, and between 2016 and 2021, it was 2.65 (StatsSA 2021). This article will follow a qualitative exploratory, descriptive and contextual design, which will allow the researcher to obtain in-depth information concerning the research questions.

### Problem statement

Despite the development of a wide network of modern healthcare delivery systems throughout the world, including countries like Bangladesh, indigenous healing systems still play a significant role in delivering healthcare at the community level (Haque et al. 2018:2). However, IPs vary from country to country according to cultural preferences and perceptions on causes of infertility in women. Subsequently, knowledge of causes of infertility also differs according to the categories of the IPs, for example, faith healer, herbalist or Sangoma. Dierickx et al. (2019:15) suggested that Western treatment in Gambia is costly, inaccessible and poorly explained on causes of health challenges, and this includes infertility.

Adding to the costs, there are various Western infertility practices that women can consult to understand the causes of infertility, but underutilised because of cost, repeated appointments and poor prognosis (Babikir et al. 2021:3; Hiadzi & Boafo 2020). However, there is little information about the views of IPs on the causes of female infertility; consequently, this study seeks to explore these views.

### Objective

This study aims to explore and describe the views of IPs on the causes of female infertility in Ngaka Modiri Molema health district.

### Research question

What are the views of IPs on the causes of infertility in Ngaka Modiri Molema health district?

## Research methods and design

This study used a qualitative, exploratory, descriptive and contextual approach to understand the views of Indigenous Health Care Practitioners (IHCPs) on the causes of female infertility in Ngaka Modiri Molema health district. The approach was to enable the researcher to obtain detailed and factual views from IHCPs regarding the causes of infertility in women.

### Study setting

The study took place in Ngaka Modiri Molema, North West Province, one of the most rural provinces in South Africa. This district is a rural area with 28 wards consisting of 102 villages and eight 24-h community health centres providing services to the community. The setting for this study was natural and an uncontrolled real-life situation.

### Population

The study population entails individuals to whom the researcher can gain access to and who have the appropriate knowledge and experience about the research question (Holloway & Galvin 2016:143). In this study, the population was all IPs who managed infertility and were members of the North West Dingaka Association in Ngaka Modiri Molema health district.

### Sampling

In this study, the sample was IPs who managed infertility in Ngaka Modiri Molema health district and who consented to participate in the study, as aligned with Brink, Van Der Walt and Van Rensburg (2016:116).

#### Sampling technique

The use of the non-probability sampling approach was because the researcher had to select participants who know the most about the phenomena. The researcher followed the purposive sampling technique, in which the selected participants were chosen for the specific purpose of having knowledge about the question at hand (Holloway & Galvin 2016:143). In this study, the researcher contacted Dingaka (Indigenous healers’) Association where the chairperson identified the experts in the management of infertility and who would consent to participate in the study.

#### Selection criteria

Inclusion criteria determined whether an individual could be a member of the population with specified characteristics (Polit & Beck 2017:366). In this study, inclusion criteria included IPs who assisted women with infertility problems and who consented to participate in the study.

### Data collection

Aligned with Polit and Beck (2017), data collection is a process described as follows:

The collection of the information was through semi-structured individual interviews using an audio recorder with informed consent from the participants. The researcher used techniques such as probing, clarifying, reflecting and paraphrasing during face-to-face interviews to achieve the main objective of the study. The individual interviews were conducted in the participants’ homes, in a quiet room without disturbance. Field notes on non-verbal communication cues were taken. Data collection continued until there was no new information emerging from the participants. (p. 89)

### Data analysis

Data were transcribed verbatim, and then according to Creswell (2014:248), the following eight steps were followed:

- Capturing the whole sense, the independent coder listened to the recordings and repeatedly read all the transcripts to gain a thorough understanding, and wrote down ideas as they came to mind.
- The research team as well as the independent coder went through the interviews that were direct to the point. In the process, thoughts that came to mind were written in the margins. A consensus discussion took place where the findings were confirmed.
- Subsequently, there was a recording of all the emerging topics, with similar topics grouped together. The independent coder drew columns, and the topics were arranged into major, unique topics and leftovers.
- The independent coder re-examined the formulated list of topics and compared it against the original data. There was documentation of the acronyms of topics into codes, followed by recording these next to the correct segments of text to verify if new codes and categories emerged.
- The independent coder established the most descriptive words for the topics and organised them according to the way they related to each other. The list of categories was decreased by grouping them into major topics that related to each other. The researcher drew lines between categories to indicate interrelationships.
- The data material suitable to each category was assembled, and the researcher performed a preliminary analysis.
- The existing data were re-coded.

### Ethical considerations

Ethical clearance was requested and obtained from the North-West University’s Health and Research Committee (Reference NWU-00689-17-A9) prior to the commencement of data collection. All information gathered remained safe and was not shared with other parties to ensure confidentiality. The researcher used numbers to identify the participants instead of names to maintain anonymity during the study.

### Trustworthiness

The five criteria of Lincoln and Guba upheld trustworthiness, namely, credibility, dependability, confirmability, transferability and authenticity (Lincoln & Guba 1985). The interviewers prolonged engagement with the IPs to gain an in-depth understanding of their views on causes of infertility in women, maintaining credibility. The researcher elaborated on the research process carried out to ensure there could be a replication of the same study. The recordings, transcripts and field notes were safely stored with password protection in a computer to enable an enquiry audit; these measures ensured the dependability of the data. In this study, a confirmability audit, triangulation of literature sources and a consensus discussion between the researcher and the independent coder ensured the confirmability of the findings. Transferability was ensured in this study’s results to other settings by providing a thick description of the study’s setting, the participants and the research method used. The attaining of authenticity was through the recorded interviews and the transcriptions of collected data. One of the research supervisory team members, who has command of the language, listened to the recordings and confirmed the transcriptions.

## Findings

The basis for the results was the views of indigenous healthcare practitioners on infertility. Table 1 presents the demographic characteristics of the IPs in this study.

**TABLE 1:** Demographics of participants.

Number	Gender	Age in years	Occupation
IP 1	Female	65	Pensioner
IP 2	Female	47	Unemployed
IP 3	Female	63	Pensioner
IP 4	Female	65	Pensioner
IP 5	Female	50	Domestic worker (cleaner)

IP, indigenous practitioners.

### Themes and sub-themes

Three main themes emerged from the data analysis process: (1) history taking regarding infertility, (2) treatment of infertility and (3) holistic care of infertility, which describes the views of IPs managing infertility in women in Ngaka Modiri Molema health district (see Table 2).

**TABLE 2:** Themes and sub-themes.

Themes	Sub-themes
History taking regarding infertility	Divination: an indigenous diagnostic method
Treatment of infertility	Plant medicine and ritualistic treatment Massage of the abdomen and the uterus area
Holistic care of infertility	Support for the women

#### Theme 1: History taking regarding infertility

In the process of consultation, the IP is guided by the spiritual world and the ancestors to identify the root cause of the problem and the treatment that should be given. During the consultation, there are questions posed to the woman, or the IP explains what she sees or hears from the ancestors with confirmation from the woman, while some IPs throw bones to detect the problem and determine treatment. Through the visions, the woman was often treated or advised on the illness that has not shown any manifestations.

IP 2 alluded:

‘… sometimes infertility can be due to miscarriage and repeated illnesses of the reproductive system that were not properly treated. The spirit can also be revealed if the woman had multiple partners and one of them was a widower who contributed to her repeated STI leading to infertility.’ (IP 2, Female, 47 years old)

IP 5 added:

‘Before the conversation starts the ancestors showed me that her problem started after they moved to their own house as the house was not properly cleansed from the evil spirits that contributed to her irregular and scanty menses. I threw the bones and noticed that her underwear is missing, and she is not aware, which was purposefully taken by witches to prevent conception.’

IP 4 mentioned:

‘The woman was given traditional medicine by her friend which was very strong and not meant for women therefore, it contributed to her continuous heavy menses and infertility.’ (IP 4, Female, 65 years old)

In the process of history taking, the IP connects with the woman’s ancestors and requests their protection and guidance.

**Sub-theme: Divination: An indigenous diagnostic method:** Divination is the technique used during consultation through which the IP relays the information from the ancestors to the woman. Divination is a ritualistic process in a spiritual perspective where the ancestors of the patient are consulted to disclose what is the cause of the illness. It is widely used as a diagnosing technique to seek answers by African community members, such as barren women and people with odd diseases who suspected their enemies arranged such sufferings to try to harm them through witchcraft (Abukari, Issah & Adam 2022:422). The IPs will have symbolic visions revealing the illness and the treatment.

IP 3 attested:

‘I consult with the spirit world by listening to what went wrong and how that can be corrected and I ask her to explain her challenges. The treatment will be based on what I see and heard from above.’ (IP 3, Female, 63 years old)

IP 5 said:

‘In some instances when I pray the cause of the problem is revealed to me and will know exactly what to do, e.g. the woman has weak eggs, then I will carefully give medication to strengthen the eggs.’ (IP 5, Female, 50 years old)

The IP clearly explains that expected of the woman, and the request should be carried out appropriately to promote blessings from ancestors.

#### Theme 2: Treatment of infertility

The study revealed that IPs in this study utilise two main approaches for the treatment of infertility depending on the diagnosis. If infertility is found to have a spiritual cause, the practitioners will follow a ritualistic approach to healing and use plant medicine in the process. While if the problem is more physical, the treatment approach will be massaging of the abdomen and the uterus area.

**Sub-theme: Plant medicine and ritualistic treatment:** Treatment is any form of intervention used by IPs to manage healthcare problems, including infertility, and can be in the form of plant medicine, water, rituals or prayer. The treatment options depend on guidance from the spirit world and instructions on how it should be carried out. Indigenous remedies for infertility are made of special plants and plant extracts that have a positive effect on the reproductive organs, hormonal system and sex drive to promote conception (Kashani & Akhondzadeh 2017).

IP 1 stated:

‘I prepare the herbs to ensure that the concoctions are properly mixed and give the woman instructions on how the ritual will take place to appease ancestors. This includes slaughtering of a white chicken and bath with its blood ….’ (IP 1 Female, 65 years old)

IP 4 said:

‘Treatment depends on the cause of infertility, if is due to curses a ritual is necessary and uterus that is not well positioned (retroverted uterus) needs massage and some medications.’ (IP 4, Female, 65 years old)

**Sub-theme: Massage of the abdomen and uterus area:** Massage can be used as a method of diagnosing or correcting the health problem. The IP during history taking might advise the women to use certain medication to massage first or later, which can be once off or ongoing process.

IP 3 added:

‘Firstly, I massage the woman to see if I can identify something that may be of significance to infertility such as the uterus that is hard and feels swollen (uterine fibroids). Then through massage I can tell that there is a mass growing inside.’ (IP 3, Female, 63 years old)

IP 2 said …:

‘I massage the woman to check how and where is the womb, is it painful during massage then will decide if is for massage only or she needs pots (medication that was boiled) to clear what is causing pain.’ (IP 2, Female, 47 years old)

#### Theme 3: Holistic care of infertility

The IPs apply a holistic approach that encompasses the mind, body, family and environment to achieve the optimal results of treatment. The woman would also receive help with other health problems that are asymptomatic, and this can include the partner to improve treatment outcomes.

IP 2 explained:

‘During consultation I don’t only attend to the problem on hand, the woman might have other challenges that she is not aware of that contribute to the problem. In the post-natal period, the women are given medication that heals the uterus, and some rituals are performed for the baby.’ (IP 2, Female, 47 years old)

IP 1 stated:

‘I assisted the lady who was bewitched by her mother-in-law to prevent her to conceive. Her problem was continuous irregular menses I helped her to move out of that house, control her menses, performed rituals and she fell pregnant few months later.’ (IP 1 Female, 65 years old)

The IPs are trusted as they can also foresee challenges before manifestations and manage accordingly.

**Sub-theme: Support for the woman:** Infertility is a reproductive health problem that affects couples, but women are the ones stigmatised and blamed for the inability to conceive. Therefore, IPs offer counselling sessions and invite the male partner for dual assistance and sometimes the family, depending on the situation at hand.

IP 1 said:

‘I requested the woman to come with her partner in order to explain the treatment process and request them to spend most of the time together until the treatment course is finished.’ (IP 1 Female, 65 years old)

IP 5 stated:

‘Some women will be rejected by her in-laws and in that case, I visit the parents to explain how the lady should be treated and encourage the partner to support her.’ (IP 5, Female, 50 years old)

Women prefer to consult with IPs because of continuous support post-delivery and all other general life problems because of affordability and accessibility.

## Discussion

Infertility, in African society, often has a link with supernatural and spiritual causes, which makes the use of faith healing or consultation with IPs a first-choice decision. Therefore, the use of indigenous medicine goes beyond its medicinal effect, as per tradition and culture (James et al. 2018:5). According to the findings, the spiritual world guides the IP, during history taking, in relation to the root of the problem and treatment. The women can verbalise their problems, or the IPs can throw bones to explore the problem. Prophecies can also describe the problem and the causes, and the assistance for the women. People consult IPs worldwide for all health problems, including infertility. Despite the availability of modern treatment methods, some women still trust and consider that the treatment for infertility depends on indigenous methods (Patil & Udgiri 2019:228). Treatment of infertility differs from woman to woman, as per individual causes and guidance from the ancestors. The findings of this study describe that IPs possess indigenous health knowledge which they use in the diagnosis and treatment of infertility. Additionally, IPs holistically explore all possible causes of infertility and through visions prepare indigenous medicine, prescribe rituals and use massage to restore fertility, as per guidance from above. It has been reported that indigenous healing practices are the preferred forms of therapy, particularly in rural sub-Saharan Africa, not only because of their availability and affordability but also because they cater to the cultural needs of healthcare users (James et al. 2018:5). This correlated directly with the findings of this study because the IPs do not only provide physical therapy but also offer spiritual healing through rituals and the use of prayer. Massage is seldom mentioned in literature as an African therapeutic practice, and it is mainly attributed as an Asian healing practice (Benedict 2014). This study highlighted that IPs would give women massage as the only therapy to facilitate conception in cases where the uterus is twisted (retroverted).

The holistic approach that the IPs use encompasses the spiritual, psychological and social well-being of a woman. The findings affirmed that IPs use prayers to gain insight on the causes of infertility and provide counselling during the process of treatment. Considering psychological perspectives, women undergoing infertility usually suffer from depression, stress and anxiety attributed by lack of support from significant others, and hence, IPs offer continuous support (Tanywe et al. 2018:1773). The literature attested that some women with infertility prefer to consult with IPs as they provide continuous holistic care and support throughout pregnancy, post-natal period and other subsequent pregnancies.

## Limitations

A major limitation of this study was that the sample was small, although in-depth interviews revealed rich data. To compensate for the smaller sample size, a literature integration was done to confirm the findings. Some of the participants were not willing to talk freely because of fear that the researcher was taking their knowledge for her own benefit. In the process of the interviews, one participant alluded that she could not tell me the names of the medication, as the researcher required training to learn them.

## Recommendations

Based on the findings of this study, nursing education should ensure that topics on human reproduction and related indigenous practices are included in the midwifery module to equip students on indigenous practices concerning infertility. There should be future research conducted on the effectiveness of indigenous practices on infertility.

## Conclusion

The objective of this study was to explore and describe the views of IPs on the causes of female infertility in Ngaka Modiri Molema health district. Indigenous practitioners are the main sources of help in the management of health problems, including infertility. The findings revealed that causes of infertility vary per individual, with treatment options based on such causes. Most indigenous African societies consider that supernatural forces trigger certain illnesses, which Western practice cannot treat, necessitating an indigenous practice for treatment.
